# Determinants of exports performance: Evidence from Indonesian low-, medium-, and high-technology manufacturing industries

**DOI:** 10.1371/journal.pone.0296431

**Published:** 2024-01-02

**Authors:** Rossanto Dwi Handoyo, Kabiru Hannafi Ibrahim, Yessi Rahmawati, Faizal Faadhillah, Keiichi Ogawa, Deni Kusumawardani, Kok Fong See, Vikniswari Vija Kumaran, Rachita Gulati

**Affiliations:** 1 Department of Economics, Faculty of Economics and Business, Universitas Airlangga, Surabaya, Indonesia; 2 Department of Economics, Faculty of Social and Management Sciences, Federal University, Birnin Kebbi, Nigeria; 3 Graduate School of International Cooperation Studies, Kobe University, Kobe, Japan; 4 Economics Program, School of Distance Education, Universiti Sains Malaysia, Gelugor, Malaysia; 5 Universiti Tunku Abdul Rahman, Kampar, Malaysia; 6 Department of Humanities and Social Sciences, Indian Institute of Technology Roorkee, Roorkee, India; University of Sargodha, PAKISTAN

## Abstract

This study explores the determinants of the export performance of Indonesia’s low-, medium-, and high-technology manufacturing industries by focusing on the role of raw-material imports and technical efficiency. Micro firm-level data from 2010–2015 were utilized for the analysis in this study. The stochastic frontier analysis was employed to measure technical inefficiency and to determine its effect on export performance. Our findings indicate that in all categories of industry technical efficiency, raw materials imports, foreign direct investment (FDI), location, firm size, labour productivity, and concentration of industries were significant determinants of export performance. While high efficiency increases exports in low- and medium-technology firms, exports decrease in firms with high efficiency accompanied by high imports, FDI, size, and labour productivity. Furthermore, in high-technology industries, efficiency reduces exports and again increases them when mediated by a concentration of industries and location. The empirical strategy also supports the positive effect of imports on export performance in both industries, which also aligns with decreased exports in firms with high imports accompanied by high FDI, efficiency, labour productivity, the concentration of industries, and size. To this end, the study has implications for low-, medium-, and high-technology manufacturing that are mainly concerned with increasing exports.

## 1. Introduction

Export performance reflects both the relative success (or failure) recorded by a country or business firms for exporting to foreign markets. Most countries in the world tend to avoid disruption in exports and are more focused on sustainable exports for better economic growth. This is because trade serves as an engine of economic growth and integration [1], and a country can use available trade potential to develop further [2]. More importantly, the country’s economic progress is the result of robust export performance [3–5]. Export policies have helped developing economies in achieving substantial economic growth [6]. Export activities have allowed developing and emerging economies to link with the world economy, increase their market base and economies of scale and promote the transfer of technology. Exports are key to the foreign exchange required to settle for imports, and there is a systematic link between exports and imports [5]. The competitive strength of the manufacturing sector is measured by the performance of its output and capacity to penetrate export markets [7]. In Indonesia, the overall manufacturing sector and its disaggregated constituents (i.e., low-, medium-, and high-technology manufacturing industries) have contributed to productivity and export growth. In Indonesia manufacturing industry when disaggregated into low-, medium-, and high-technology can be further defined as; the high-technology industries including chemicals and chemical products, pharmaceuticals, computer/electronic/optical products, electrical equipment, machinery and equipment, motor vehicles-trailers-semi-trailers, and other transport equipment except for ships and boats. The medium technology industry includes rubber/plastic products, other non-metallic mineral products, basic metals, other manufacturing except medical/dental instruments, and repair and installation of machinery and equipment. The low technology industry is defined as food products, beverages, tobacco products, textiles, wearing apparel, leather and related products, wood and products of wood, paper and paper products, printing and reproduction of recorded media, coke and refined petroleum products, and fabricated metal products except for weapons and ammunition and furniture. The sector accounts for a larger share of GDP [8]. It is a well-known fact that technology is an important factor in international trade [9]. There has been an enormous change in manufacturing exports owing to technological change in recent decades [10]. This demonstrates the links between export performance and technology in the manufacturing industry. However, the theoretical proposition postulates that technology plays a key role in determining trade patterns. The role of technology in accelerating the manufacturing sector has long been documented by [11, 12]. Therefore, exporting firms-specific technology has been the prime mover of manufacturing exports in developing countries like Indonesia. Likewise, expanding exports is associated with increased technology and technological transfer to exporting countries [13]. The technology-intensive manufacturing exports have grown significantly over the last decades [14]. The level of technology in the manufacturing sector has been found to affect export behaviour in Indonesia [15]. Exports from the manufacturing sector are mainly from high-technology firms [7]. Some technologies like computer-aided design (CAD) may not suit industries manufacturing for the domestic market [16], but can invariably boost firm manufacturing for export. There is a need to look at determinants of export performance in Indonesia’s manufacturing as it relates to firms’ level of technology adoption. This is important because manufacturing firms vary significantly in the way they adopt technology. This led to different manufacturing industries being categorized based on low, medium, and high levels of technology adoption.

The Indonesian government seeks to encourage increased productivity in the manufacturing sector to optimize the potential of the export market. In Indonesia, between 2011 and 2017, net exports have consistently been increasing [17], while in recent times, the performance of net Indonesia’s exports, especially in textile/apparel and many other industries, has been declining [18]. To live in the world of competition, exporting firms in Indonesia need to restrengthen the performance of their exports. As argued by [19] exporting firms in advanced market settings need to maintain high quality standards and technical safety in products. With the current trend, and to improve exports, there is a need to critically scrutinize the determinants of manufacturing export performance, especially while disaggregating manufacturing into low-, medium-, and high-technology industries.

Exports and export performance are influenced by numerous factors, including imported raw materials, firm size, FDI, efficiency, labour productivity, location, and industry concentration, among others. Indonesia and other developing countries are highly dependent on imported raw materials and other intermediate inputs. Raw material imports are the main source of manufacturing sector productivity and a determinant of a company’s tendency to increase productivity, which in turn influences exports, especially for small companies [20]. Higher access to raw material varieties has a larger impact on exports than an increase in import volume [21]. As an important input for production, a critical area of concern for importing raw materials is that it makes production costs more sensitive to international price volatility [22]. With the increase in the exchange rate, the rising import of raw materials may deteriorate productivity [23]. Apart from the problem of importing raw materials, another issue that stands out for the manufacturing industry is company efficiency. Companies with high efficiency can enlarge their market, create international market links, and take advantage of technology transfer [24]. Much empirical evidence has demonstrated the positive link between export and a company’s efficiency. For instance [25], observed that more efficient firms are export-oriented than less efficient firms.

The nexus between export and firm size has been well documented in the literature. A non-linear nexus exists between export and firm size [26, 27]. This demonstrates that the gainful effect of a firm’s size on export only appears at a certain level of threshold. Another important determinant of export is the existence of foreign ownership through FDI. Foreign firms’ exports can outweigh domestic firms’ exports because they use up-to-date technology and managerial skills and are more efficient than local firms. Through the spillover effect, FDI can improve a country’s efficiency and productivity level. Empirical studies by [28–30] have documented the positive effect of FDI on exports. Theoretically, a self-selection theory has established the link between export and labour productivity in which firms partake in the export market because of their high productivity. Another theory that explains this nexus is the learning-by-doing theory. The theory posits that companies partaking in the export market will become more efficient and more productive. An increase in productivity while exporting could result in technology adoption, innovation, and increased competitiveness [31].

The main contribution of this study lies in an examination of the determinants of export performance in Indonesia’s manufacturing industry while disaggregating the industry into low-, medium-, and high-technology industries. This is important because firms’ level of technology adoption has an even greater influence on exports and can account for different export performances between firms. Additionally, the study used a dataset that virtually covered all manufacturing firms in Indonesia and analysed the technological structure of export performance. An exploration of this nature as it relates to the level of technology adoption has not been largely investigated by recent literature. Studies in the context of Indonesia have mainly focused on the export performance of textile industries [18], manufacturing industries [3, 15, 32–35], vanilla products [36], disaggregated firm-level data [21]. Some other recent studies [37, 38] have also evaluated the qualitative aspect of export and emphasized on trade diversification strategy, energy consumption, and economic growth. With, therefore, the present study aims to examine the determinants of export performance in Indonesia’s low-, medium-, and high-technology manufacturing industries. This is important because the industry is, export-oriented industry that employs many skilled, semi-skilled, and low-skilled labour in Indonesia. The industry’s large contribution to employment has not yet matched with its international competitiveness. This is because in the international market, the competitive position of this subcomponent of the manufacturing industry is less promising. Despite the low competitive position of this category of industry, it remained the major contributor to economic growth. This underscores the importance of this sector to the national economy. The study also focused on the role of raw materials in influencing export performance. This is because Indonesia’s low-, medium-, and high-technology industries heavily relied on a high proportion of imported raw- materials. This has posed a threat to Indonesia’s balance of payment (BoP), as the industry is currently contributing lower exports. Studies have not been conducted to explore the determinants of export performance in Indonesia’s manufacturing industry by disaggregating the industry into low-, medium-, and high-technology industries. The study also aims to examine the indirect channels through which the determinants of exports affect export performance. With this in mind, therefore, the study will add to the literature by examining the effect of technical efficiency, raw material imports, foreign ownership, firm location, firm size, labour productivity, and industry concentration on export performance over the period 2010–2015.

This study is organized into five sections. Section 1 introduces the paper, and section 2 presents the literature review. Section 3 discusses the methodology. Section 4 presents the study findings, and finally, section 5 concludes the paper and provides policy recommendations.

## 2. Literature review

There are many studies conducted on the determinants of export performance in different countries (developed and developing). For instance, in the case of Indonesia’s textile and apparel industries, a recent study by [18] found a significant positive effect of industries’ imported inputs on the performance of their exports. Although the effect is found to be larger on the apparel industry than on the textile industry, this is corroborated by the fact that access to foreign raw materials tends to promote exports. This imported input influence on export performance will not guarantee liberalization of imported input in Indonesia. To protect local industries, there is a need for certain restrictions, especially on input which will pose a threat to local industries. Similarly [32], report that the concentration of industry, FDI, raw material imports, technical efficiency, and firm size assert a positive and significant effect on manufacturing exports. This implies that firms with more foreign ownership, high labour productivity, large firm size, high technical efficiency, high concentration of industries, high imported raw materials, and location of industries have high export performance. From this finding a concerted effort is needed to increase firms’ export performance by way of introducing programs related to export, enhancing research and development to increase the skilled workforce in the export sector, protecting local forms, and reducing bureaucracy attached to the inflow of foreign investment among many others. [5] observed a long-run nexus between export performance and capital and intermediate goods imports in the case of Pakistan. His finding suggests a long-run export elasticity of 37% with 24% and 16% contribution to exports resulting from raw material and capital goods imports. Despite that, the influence of the total imports on exports is not large, it underscores the importance of capital and intermediate goods imports as critical factors in the analysis of export performance. A study by [39] revealed that gross domestic product (GDP), foreign direct investment (FDI), and official development assistance (ODA) significantly promote export performance, while the labour force and inflation rate negatively affect export performance in Somalia. Although for many years the Somalian economy has been negatively affected by conflict, most variables seem to have positively promoted export performance. This finding underscores the importance of hat gross domestic product (GDP), foreign direct investment (FDI), and official development assistance (ODA) in promoting foreign trade. While conceptualizing and measuring export performance differently with geographical context [13], reports a significant positive effect of past export performance in Caribbean English-speaking countries. This finding is also interceded by the firm’s ability and adoption strategy. Satisfactory experience in past export performance has been found to affect a firm’s commitment to improving current export performance. To this end, maintaining export performance and competitiveness by manufacturers requires global initiatives. In a study by [40], the possibility of agro-processing companies participating in exports is influenced by expenditure on imported raw materials, the firm’s age, firm size, infrastructure, and investment in technology. Their finding further observed expenditure on imported raw capital goods to be positively linked to forms of export intensity. This implies that firms in areas with high infrastructure development, large size, high managerial skills, and high age are more prone to engage in export activities than otherwise.

Other studies have specifically focused on the link between exports and raw material imports. These include [20] who affirmed that, in terms of productivity, size, and capital intensity firms with experience in foreign markets perform differently. Firms engaged in both exports and imports perform significantly while firms that only engaged in imports perform higher than firms that engaged in exports only. By engaging in trading activities there exists an importers’ premium which supports the self-selection hypothesis. [21], revealed that raw material imports add to the export basket of the Swedish and Indonesian manufacturing industries. Increased access to inputs promotes imports and impacts on export performance and when the imports were from developed countries the impact is even larger. This corroborates with technological and product quality instilled in inputs that influence Indonesia’s export performance. [22] have found an increase in tariff reduces Indonesia’s textile and clothing export to the US while raw material import promotes export. To further promote exports, they saw the need to improve market access, reduce manufacturing costs, input management, and improve production capacity. [41] estimates no significant impact of import on productivity and imports sizeable and reasonable impact of between (2% to 5%) for non-electrical firms. In the case of Ethiopia, a study by [42] has found that export intensity, classification of industry, and ownership structure mediate with the nexus between performance and raw materials import. Furthermore, the nexus between FDI inflow and raw materials import was larger when the firms had high exports and joint ventures. This suggests that firms with these characteristics can engage in raw materials exports. [4] observed that to increase their productivity, Thai exporting and manufacturing small and medium enterprises (SMEs) relied more on labour as against capital and there is. an increasing return to scale in Thai manufacturing and exporting SMEs to Oceania.

As reported by [35], FDI positively affects export performance in Indonesia’s manufacturing industry. Moreover, there exists strong evidence that export generates FDI in the high-technology industries, physical capital-intensive industries, and human capital industries. A contradictory finding has been found by [43] in the case of the Indian pharmaceutical industry. In a study by [44], foreign technology has been found to promote export performance in 141 Chinese manufacturing firms. The performance of exports varies depending on the sources of external technology. The performance of firms that used foreign technology in manufacturing outweighs those that used locally developed technology. Similarly, in a study by [15], technology has been found to affect Indonesia’s manufacturing exports. In addition, the study further observed that cost-related factors also influence export behaviour.

[45] has found that GDP and population growth positively affect Ethiopia’s flower export performance, while the exchange rate negatively affects the dynamic of export performance. Hence supply-side and domestic factors were the prime determinants of Ethiopian export performance and this required expanding human capital, promoting GDP growth, and maintaining a stable exchange rate policy. A study by [36] revealed that gross domestic product (GDP), domestic production and consumption were significant determinants of Indonesia’s vanilla export. With these factors influencing export performance, improved technology, knowledge adoption, marketing management, and product development.

[28] examined the determinants of export participation in Thai manufacturing SMEs in which financial institutions like export-import banks and the department of international trade and promotion were found to be significant factors affecting export intensity and export participation. Domestic and foreign banks were not significant factors affecting export intensity and export participation in manufacturing SMEs. Export is lesser for SMEs who source funds from family and friends. This finding demonstrates the role of foreign ownership in promoting export intensity and export participation and that manufacturing SMEs are likely to have lesser export intensity and export participation than big manufacturing enterprises. In the case of the Kenyan manufacturing industry [7], compared the efficiency of exporting and non-exporting firms in which he observed exporting firms to be more efficient than non-exporting firms. Capital-intensive firms attract more markets outside the African continent than firms that are not intense in human and physical capital. Furthermore, firm size is an important factor in export performance and there is a learning effect in export participation, especially with developed countries as against developing countries. Taking destination into account has therefore brought to light more understanding of the t nexus between exports and efficiency. In the case of the Chinese clothing industry, a significant U-shaped nexus exists between efficiency level and export orientation, as observed by [46]. With a large extent of export orientation and experience and high sales in the domestic markets industry, specific effects can explain clothing firms in the Chinese Guangdong province. [24] observed a positive link between export revenue and technical efficiency in the Indian textile industry. Their study finding revealed that the textile industry with high export revenue had higher technical efficiency while those with low revenue recorded a low level of technical efficiency. Additionally, as per the size of firms, export performance and technical efficiency do vary significantly. [34] applied a fixed effect model and analyses the determinants of export performance in the Indonesian manufacturing sector. Finding indicate that firm size, export diversification, capital intensity, and technical efficiency significantly affect export performance. Export participation is consistently linked to the technical efficiency of Indonesia’s textile industry, as observed by [47]. Absorptive capacity and export have been found to influence the efficiency levels in the Indonesia manufacturing industry [23].

[48] observed that Portuguese wine firms’ size promotes export performance, while there is no strong evidence for the effect of efficiency on export performance. The finding also indicates that export performance responds positively to age which is also higher for smaller and bigger firms. Studies by [49–51] observed that firm size affects companies’ decision to export in Botswana and the MENA region. Empirical findings by [52–54] established the link between labour productivity and exports. In the case of Australia, industrial location has been found to increase export performance in a study by [55]. Over the period 1995–2019 [56], analysed the potential, determinants, and efficiency of Nigeria’s agriculture commodity exports to the EU. Their findings as obtained from the extended gravity model and SFA revealed GDP, exchange rate and new member states negatively affect agri-food exports to EU countries. Similarly [1], report that economic size (GDP), ECOWAS membership, EU membership, importers’ population, and contiguity promote agri-food exports to 70 of Nigeria’s major trading partners. Border countries and larger economies have been found to remain the potential markets for Nigeria’s agri-food exports. Another study by [57], indicates that the economic size of importing countries, the Belt and Road Initiative (BRI), the Chinese language, and the common border promote Chinese agricultural exports. On the other hand, findings also indicate exports are adversely affected by distance, land-locked, currency depreciation, and China and its trading partners’ GDP per capita. [58] revealed that Indonesia’s per capita GDP, importer GDP, colonization, exchange rate, and membership of WTO assert a significant positive influence on palm oil downstream exports. A negative influence has been reported in the case of geographical distance, Indonesia’s GDP, importer’s GDP per capita, and landlocked. In the case of Ghana [59], examined the potential bilateral export gap with 61 major trading partners for the period 2000–2018. Findings indicate that there exists an untapped export potential in major export market destinations. However, low credit facilities to the private sector, lack of infrastructure, and tax burden of export destinations significantly hinder the exploitation of potential exports. [60] reports India has exploited fully the efficiency of its exports to trading partners under free trade agreements (FTAs). Additionally, trading blocs such as ASEAN, MERCOSUR, and SAFTA stimulate India’s export efficiency.

From the foregoing literature, it is evident that an examination of the determinant of export performance of manufacturing firms while disaggregating the sector into low-, medium-, and high-technology manufacturing industries has not been investigated. This, therefore, represents a gap in literature with which this study aims to fill.

## 3. Method and data

This study uses firm-level data obtained from the annual survey of manufacturing establishments of the central bureau of statistics of Indonesia (BPS). We used a quantitative analysis with a panel data set that is believed to provide more degrees of freedom. The data comprised 5,886 low-level technology firms, 1,374 medium-technology firms, and 684 high-technology firms. The study highlights the differences in the export performance of high-, medium-, and low-technology industries over 2010–2015. The choice for this period depends solely on data availability as there is no available to go beyond this period. We adopt the stochastic frontier model to measure the efficiency of Indonesia’s manufacturing firms. This is important because the model capture can capture a firm’s technical inefficiency and heteroskedasticity. Additionally, the method can provide an unbiased estimate of frontier parameters, robust to skewness of the residual and outlier and is the most desirable model compared to OLS [60]. More importantly, the stochastic frontier can address the endogeneity problem with the specified model.

The technique for the analysis in this study involved two stages. We first estimate the value of the company’s efficiency by reckoning the parameters of the production function using the "Frontier 4.1" software developed by [61]. Efficiency estimates can be best handled using stochastic frontier analysis with a specified production function. Second, we determine the factors that influence the company’s export probability by using logistic regression analysis. The logit model is applied to a non-linear regression model whose dependent variable is categorical with values of 1 and 0 (binary). In this study, the logit model becomes suitable because export performance is measured using dummy variables with 0 and 1.

The value of a company’s technical efficiency lies between 0 and 1. The closer to a value is to 1, the more efficient the company is [61]. Following the work of [62], the technical efficiency variable is obtained from the stochastic frontier analysis (SFA) transcendental logarithmic (trans log) and Cobb‒Douglas production function. Eq (1) is the specifications for the general model of the production function which is expressed as follows:

$$lnY_{it}=\beta_{0}+\beta_{1}(lnK_{it})+\beta_{2}(lnL_{it})+\beta_{3}(lnM_{it})+\beta_{4}(lnE_{it})+v_{it}-u_{it}$$ (1)

where *i* is the individual unit, in this case, firms in the industry, *t* is the time of the observed value, *ln* is the natural logarithm, *Y* is the total firms’ output level, *K* is the capital stock used by the firms within an industry, *L* is the labour used in the production process, *M* is the raw material used to produce *Y* unit of output, *E* is the amount of energy used in the production, and. *β*′_*S*_ are the parameters to be estimated. The *v*, is the error term that is symmetrical and assumed to be correlated with random factors not under the influence and control of firm *i*. The *u* represents the non-negative random factors (i.e. error terms component) that are under the control of firm *i*. This also signifies the firm’s productive technical inefficiency in relation to the stochastic frontier quantities as observed in the explanatory variables and symmetrical error terms (*v*). However, the random symmetric error terms are assumed to be independently and identically distributed i.i.d i.e. *N* (*0*, *σ*^*2*^) and are also independent of *u*_*i*′*s*_. Any discrepancy between the firm’s potential and actual output could result in an error having a value greater than zero (0) but less than one. A zero value of the error indicates no difference between the actual and potential output. This notion has been contrary to deviations in random disturbance terms where the deviations are a result of factors that are under the control of the firm [63].

The assumed theoretical specification of the transcendental logarithmic (trans log) model for low, medium, and high technology manufacturing export used in this study is expressed as follows:

$$lnY_{it}=\beta_{0}+\beta_{1}(lnK_{it})+\beta_{2}(lnL_{it})+\beta_{3}(lnM_{it})+\beta_{4}(lnE_{it})+\frac{1}{2}\beta_{5}{\left(lnK_{it}\right)}^{2}+\frac{1}{2}\beta_{6}{\left(lnL_{it}\right)}^{2}+\frac{1}{2}\beta_{7}{\left(lnM_{it}\right)}^{2}+\frac{1}{2}\beta_{8}{\left(lnE_{it}\right)}^{2}+\beta_{9}(lnK_{it})(lnL_{it})+\beta_{10}(lnK_{it})(lnM_{it})+\beta_{11}(lnK_{it})(lnE_{it})+\beta_{12}(lnL_{it})(lnM_{it})+\beta_{13}(lnL_{it})(lnE_{it})+\beta_{14}(lnM_{it})(lnE_{it})+\beta_{15}t+\beta_{16}t^{2}+\beta_{17}(lnK_{it})(t)+\beta_{18}(lnL_{it})(t)+\beta_{19}(lnM_{it})(t)+\beta_{20}(lnE_{it})(t)+v_{it}-u_{it}$$ (2)

In Eq (2), all the variables are as defined in Eq (1) except that, Eq (2) is an expanded version of Eq (1) that includes interaction terms necessary to estimate the value of firms’ technical efficiency. Given the stochastic frontier function of trans log nature and the *v*_*i*_ and *u*_*i*_ density function we applied the maximum likelihood estimation method to estimate the parameters of Eq (1). This has been reported in Table 2. The difference between frontier output and technical efficiencies can be substantiated through the use of stochastic frontier production which can also determine whether such difference is due to random factors or firm-specific factors. Firms whose production level lies on the frontier are considered technically efficient while those that operate below are technically inefficient.

Given the technology available, a firm’s technical efficiency is defined as the ratio of observed output to the frontier output. Therefore, the company’s technical efficiency is calculated using as expressed in Eq (3).


$${Eff}_{i}=\frac{Y_{i}}{exp(x_{i}\beta )}=\frac{exp(x_{i}\beta -u_{i})}{exp{(x}_{i}\beta )}=\exp\left(-u_{i}\right)$$ (3)

Where; *Eff*_*i*_ is firm *i*′*s* technical efficiency, *Y*_*i*_ is the firm *i*′*s* output, *exp* (*x*_*i*_*β*) is the frontier output. The difference between the firm’s output and the frontier output resulting from technical efficiencies is explained by *y* ratio given as;

$$y=\frac{\sigma^{2}u}{\sigma^{2}}$$ (4)

Eqs (1) and (2) are the stochastic frontier models for Indonesia’s manufacturing firms’ output. Therefore, we can specify the inefficiency model by expressing the inefficiency influence of *u*_*i*′*s*_ which is a function of many sets of explanatory variables. Therefore, the inefficiency component of the stochastic frontier model is given by:

$$u_{it}=X_{it}ʎ+\gamma_{it}$$ (5)

Where; *u*_*it*_ is a measure of technical inefficiency of firm *i* at time *t*, *X*_*it*_ is a vector (1×*m*) of independent variables believed to have influenced the firm’s technical inefficiency, ʎ is the vector (1×*m*) of parameters to be estimated, and *γ*_*it*_ is an approximation of the normal distribution with mean zero (0) and variance *σ*^*2*^ i.e. *N* (*0*, *σ*^*2*^) [64].

To investigate the determinants of export performance in the Indonesian low-, medium-, and high-technology industries, we estimate the following logit models:

$$L_{it}=ln\left(\frac{P_{it}}{1-P_{it}}\right)=\beta_{1}+\beta_{2}{Eff}_{it}+\beta_{3}{Im}_{it}+\beta_{4}{FDI}_{it}+\beta_{5}{Loc}_{it}+\beta_{6}{Size}_{it}+\beta_{7}{LP}_{it}+\beta_{8}{HHI}_{it}+u_{it}$$ (6)

In Eq (6), export performance (*L*) is the logit, *P*_*it*_ is the probability that a given firm does export, 1−*P*_*it*_ is the probability that a given firm does not export, $\frac{P_{it}}{1-P_{it}}$ is the export performance which is measured as the ratio of the probability that a given firm does export and the probability that it does not export. This is adopted following the work of [48] who considered export performance as a binary variable. The value of a company’s technical efficiency (*Eff*) lies between 0 and 1; the closer the value is to 1, the more efficient the company is. This variable is expected to assert a positive influence on export performance. The raw material import (*Im*) variable is measured by a dummy variable, with a value of 1 indicating that the company imports raw materials and 0 otherwise. This variable is expected to assert a positive influence on export performance. The FDI variable is a dummy variable with a value of 1 if the company receives foreign investment of more than 10% of the company’s total capital and 0 otherwise. Depending on the nature of FDI this variable is expected to assert either a positive or negative effect on export performance. The location (*Loc*) variable is a dummy, with a value of 1 if the company’s location is on Java Island and otherwise. This is also expected to assert either a positive or negative effect on export performance. This variable is important because in terms of market size Java Island is dominant with about 60 percent of the Indonesian population. The island has remained the major source of manpower required in the manufacturing industry. Most business firms are looking for a domestic market and available manpower on the island. The cost of transportation and logistics are low in the island as compared to what is obtained in Java island. More importantly, from the perspective of the industrial location of the manufacturing setting, most manufacturing firms are located in Java Island with about 80 per cent located in the island. (80%). We, therefore, believe that adding dummy variables of the location of the firm in Java will contribute to the literature due to the agglomeration effect. This will also reveal the existence of supply chain factors among the manufacturing firms. Another reason for considering Java island is that, the existence of high and sophisticated infrastructure like toad network, rail lines, sea and air ports, electricity and other public utilities. The *Size* is measured by the number of employees with a value of 1 if a company has 100 or more labourers and 0 otherwise and is expected to increase export performance. The productivity of labour is measured by dividing the value of the company’s output and the amount of labour with a positive a priori expected sign. The Herfindahl-Hirschman-Index (*HHI*) is used as a proxy of the concentration of industry, which is calculated with the following formula:

$${HHI}_{it}={\left(\frac{Q_{it}}{Q_{jt}}\right)}^{2}$$ (7)

The *Q*_*it*_ is the total output of firm _*i*_ in year *t*_,_ and *Q*_*jt*_ is the total output in industry *j* in year *t*. This variable is also expected to positively affect export performance. We opted to use dummy variables instead of the actual value of most of the study variables to improve the reliability of our empirical findings. This again contradicts and differs from most existing studies that used the actual numerical values of most determinants of export performance. The use of dummies in this study will help increase the model fitness rather than the use of actual values. More importantly, we used dummies to differentiate the sample firms based on which firms export and vice versa, which firms import raw materials and vice versa etc.

We extend Eq (6) by incorporating interaction terms to measure the indirect influence of technical efficiency and raw material imports on export performance. Different model variants were used to determine the indirect effects of efficiency and imports (*Eff*×*Im*), efficiency and FDI (*Eff*×*FDI*), efficiency and location of industry (*Eff*×*Loc*), efficiency and firm size (*Eff*×*Size*), efficiency and labour productivity (*Eff*×*LP*), efficiency and concentration index (*Eff*×*HHI*), imports and FDI (*Im*×*FDI*), imports and location (*Im*×*Loc*), imports and firm size (*Im*×*Size*), imports and labour productivity (*Im*×*LP*), and imports and location of industry (*Im*×*HHI*). These estimates will help in understanding how the effect of efficiency on export performance will be different: (i) if the firm used imported raw material and vice versa, (ii) if over 10% of the firm’s capital stock constitutes foreign ownership and vice versa, (iii) if the firm is located in Java Island and vice versa, (iv) if the firm has over 100 employees and vice versa, (v) if the firm has the highest average productivity and vice versa, and (vi) if the firm is concentrated within the industrial location. Additionally, the indirect effect of imports will indicate how the effect of imports on exports can be influenced by FDI (if the firm has larger foreign capital), location (if the firm is located in Java Island), size (if the firm is a large enterprise), labour productivity (if the firm has larger average productivity), and location (if the firm is located within the industrial location). The interaction terms included in the model could be interpreted as the combined effect of the two interacted variables. It measures how the influence of one explanatory variable on the explained variables depends on the magnitude of another explanatory variable [65]. For instance, (*Eff*×*Im*) would explain how the effect of technical efficiency on export performance depends on the effect of imported raw materials. This could be interpreted as the effect of a firm with high technical efficiency and import raw materials.

## 4. Results and discussion

As part of the empirical findings in Table 1, we present the descriptive statistics of the variables used in this study. The variables used in the efficiency model and estimate of the technical efficiency were all log-transformed. In the export performance model, the variables were measured using dummies, indices, and ratio units. Based on the variables’ standard deviations, except for labour productivity, there is not much deviation among the variables involved, implying that the observed firms are more homogenous rather than heterogeneous. The observed descriptive analysis revealed that the lowest standard deviations appeared in the case of technical efficiency (*Eff*) for both the low-, medium-, and high-technology industries with corresponding values of 0.131, 0.140 and 0.160 respectively. While the highest standard deviation appeared in the case of labour productivity in all categories of industries. There is no doubt about high deviation in labour productivity considering the way it is measured in this study. Additionally, we reported the efficiency score for each category of industry in the appendix section (Appendix A1 in S1 Appendix). Over the study period, all the categories of industries had a very low efficiency score which even fell below the 50% average. This shows that all the industries are sub-optimally performing and have the potential to improve their technical efficiency which could lead to increased exports. The industry with the highest efficiency score is the medium technology industry which has a 44% efficiency level.

**Table 1:** Descriptive statistics of the variables.

Variable	Unit	Observations	Mean	St. Dev	Min	Max
**Low-Technology**						
Output (*Y*)	Log	35316	15.453	1.924	8.385	24.327
Capital (*K*)	Log	35316	13.926	2.097	4.168	26.472
Labor (*L*)	Log	35316	4.056	1.151	2.996	10.630
Material (*M*)	Log	35316	014.641	2.088	2.431	23.549
Energy (*E*)	Log	35316	11.576	2.302	20.617	20.617
Export	Dummy	35316	0.202	0.401	0	1
Technical efficiency (*Eff*)	Index	35316	0.560	0.131	0.146	1
Raw Material Import (*Im*)	Dummy	35316	0.104	0.305	0	1
FDI	Dummy	35316	0.057	0.231	0	1
Location (*Loc*)	Dummy	35316	0.842	0.364	0	1
The size of Firm (*Size*)	Dummy	35316	0.242	0.429	0	1
Labour Productivity (*LP*)	Ratio	35316	258398.8	1296789	53.311	7.10e+07
Industry Concentration (*HHI*)	Ratio	35316	0.083	13.394	0	2514.754
**Medium-Technology**						
Output (*Y*)	Log	8244	15.650	2.218	10.954	24.162
Capital (*K*)	Log	8244	14.319	2.104	5.533	26.041
Labor (*L*)	Log	8244	4.214	1.180	2.996	9.849
Material (*M*)	Log	8244	14.494	2.554	6.451	22.735
Energy (*E*)	Log	8244	12.750	2.149	4.668	22.358
Export	Dummy	8244	0.192	0.394	0	1
Technical efficiency (*Eff*)	Index	8244	0.531	0.140	0.210	1
Raw Material Import (*Im*)	Dummy	8244	0.173	0.379	0	1
FDI	Dummy	8244	0.083	0.276	0	1
Location (*Loc*)	Dummy	8244	0.824	0.381	0	1
The size of Firm (*Size*)	Dummy	8244	0.303	0.459	0	1
Labour Productivity (*LP*)	Ratio	8244	357964.8	1219487	2495.477	4.81e+07
Industry Concentration (*HHI*)	Ratio	8244	0.021	0.676	0	41.729
**High-Technology**						
Output (*Y*)	Log	4104	17.306	2.101	11.361	24.678
Capital (*K*)	Log	4104	15.736	2.232	5.768	27.510
Labor (*L*)	Log	4104	4.669	1.267	2.996	9.092
Material (*M*)	Log	4104	16.221	2.248	6.231	23.789
Energy (*E*)	Log	4104	13.451	2.244	2.326	23.478
Export	Dummy	4104	0.285	0.452	0	1
Technical efficiency (*Eff*)	Index	4104	0.437	0.160	0.078	0.972
Raw Material Import (*Im*)	Dummy	4104	0.453	0.498	0	1
FDI	Dummy	4104	0.389	0.488	0	1
Location (*Loc*)	Dummy	4104	0.904	0.295	0	1
The size of Firm (*Size*)	Dummy	4104	0.474	0.499	0	1
Labour Productivity (*LP*)	Ratio	4104	1265237	1.60e+07	234.057	8.55e+08
Industry Concentration (*HHI*)	Ratio	4104	0.187	3.050	0	141.162

Source: Authors’ computation

The estimates of the technical efficiency using the maximum likelihood method of the stochastic frontier production function are reported in Table 2. The estimates of the determinants of export performance for low-, medium-, and high-technology manufacturing are reported in Tables 3–5. As noted earlier in the previous sections, with emphasis on the role of raw materials and technical efficiency, the present study aims to empirically scrutinize the determinants of export performance in the low-, medium-, and high-technology manufacturing industries. To reaffirm the aptness of the estimated results we conducted some diagnostics checking. The estimated findings were based on three different category industries as mentioned earlier and in furtherance to that different models were estimated to ensure the robustness of the empirical findings. Consistent with [4], from the estimate of the trans-log model and based on the size of estimated parameters the exports of manufacturing industries heavily relied on labour inputs rather than capital. This implies that the industries have higher labour productivity for export than capital which suggests low technology adoption in the manufacturing processes. This also aligned with a low level of efficiency as reported in Appendix A1 in S1 Appendix.

**Table 2:** Trans log model for low-, medium-, and low-technology manufacturing industry.

Variable	Low Tech.	Medium Tech.	High Tech.
Constant	3.74***	4.99***	5.01***
	(0.1225)	(0.2645)	(0.5765)
*lnK*	0.17***	0.07***	0.15***
	(0.0131)	(0.0249)	(0.0448)
*lnL*	0.82***	0.92***	0.78***
	(0.0276)	(0.0586)	(0.0964)
*lnM*	-0.02	0.17***	0.12**
	(0.0141)	(0.0270)	(0.0571)
*lnE*	0.49***	0.13***	0.24
	(0.0126)	(0.0274)	(0.0539)
*lnK* ^*2*^	0.005***	0.006***	0.006***
	(0.0004)	(0.0009)	(0.0013)
*lnL* ^*2*^	0.03***	0.056***	0.06**
	(0.0029)	(0.0050)	(0.0094)
*lnM* ^*2*^	0.07***	0.06***	0.06**
	(0.0006)	(0.0014)	(0.0025)
*lnE* ^*2*^	0.03***	0.04***	0.03***
	(0.0007)	(0.0014)	(0.0024)
*LnK × lnL*	0.02***	0.01***	0.04***
	(0.0017)	(0.0034)	(0.0053)
*lnK × lnM*	-0.02***	-0.02***	-0.03***
	(0.0011)	(0.0020)	(0.0033)
*lnK × lnE*	-0.005***	0.006***	-0.004
	(0.0010)	(0.0020)	(0.0034)
*LnL × lnM*	-0.08***	-0.08***	-0.09***
	(0.0022)	(0.0044)	(0.0062)
*lnL × lnE*	0.0004	-0.008*	-0.01**
	(0.0020)	(0.0045)	(0.0060)
*LnM × lnE*	-0.06***	-0.07***	-0.05***
	(0.0011)	(0.0022)	(0.0041)
*t*	0.16***	0.10***	0.17***
	(0.0100)	(0.0202)	(0.0460)
*t* ^*2*^	-0.001	0.009***	-0.00
	(0.0007)	(0.0014)	(0.0029)
*lnK(t)*	-0.003***	-0.08***	0.005**
	(0.0007)	(0.0015)	(0.0024)
*lnL(t)*	0.01***	0.009***	0.02***
	(0.0014)	(0.0028)	(0.0052)
*lnM(t)*	-0.007***	0.001	-0.01***
	(0.0008)	(0.0014)	(0.0032)
*lnE(t)*	-0.0008	-0.001	-0.0008
	(0.0007)	(0.0015)	(0.0028)
sigma-squared	0.18***	0.21***	0.42***
	(0.0033)	(0.0112)	(0.0343)
gamma	0.51***	0.57***	0.60***
	(0.0061)	(0.0123)	(0.0180)

Source: Authors’ computation

**Table 3:** Export performance model for the low-technology manufacturing industry.

Variable	Model 1	Model 2	Model 3	Model 4	Model 5	Model 6	Model 7	Model 8	Model 9	Model 10	Model 11	Model 12
*Eff*	0.82***	1.082***	1.102***	1.152***	2.432***	15.032***	0.822***	0.822***	0.842***	0.832***	0.802***	0.822***
	(0.112)	(0.12)	(0.116)	(0.229)	(0.141)	(0.941)	(0.112)	(0.112)	(0.112)	(0.112)	(0.112)	(0.122)
*Im*	0.982***	2.052***	0.982***	0.982***	0.992***	0.972***	0.982***	1.032***	0.6352***	0.652***	2.782***	0.982***
	(0.042)	(0.184)	(0.042)	(0.042)	(0.042)	(0.042)	(0.042)	(0.045)	(0.106)	(0.069)	(0.379)	(0.042)
*FDI*	1.322***	1.342***	3.392***	1.322***	1.362***	1.352***	1.322***	1.422***	1.312***	1.322***	1.332***	1.322***
	(0.055)	(0.055)	(0.252)	(0.055)	(0.056)	(0.056)	(0.465)	(0.070)	(0.055)	(0.056)	(0.055)	(0.055)
*Loc*	-0.472***	-0.472***	-0.472***	-0.22	-0.462***	-0.472***	-0.472***	-0.462***	-0.512***	-0.482***	-0.462***	-0.472***
	(0.037)	(0.037)	(0.037)	(0.154)	(0.037)	(0.037)	(0.037)	(0.037)	(0.039)	(0.0337)	(0.037)	(0.037)
*Size*	1.442***	1.442***	1.442***	1.442***	3.642***	1.472***	1.442***	1.442***	1.442***	1.362***	1.442***	1.442***
	(0.032)	(0.032)	(0.0321)	(0.032)	(0.128)	(0.0322)	(0.032)	(0.032)	(0.0321)	(0.0348)	(0.032)	(0.032)
*LP*	-0.092***	-0.092***	-0.092***	-0.092***	-0.082***	0.632***	-0.092***	-0.092***	-0.092***	-0.082***	-0.062***	-0.092***
	(0.013)	(0.013)	(0.013)	(0.013)	(0.013)	(0.049)	(0.013)	(0.013)	(0.013)	(0.013)	(0.014)	(0.013)
*HHI*	0.03	0.04	0.03	0.03	0.05*	0.06**	-0.04	0.03	0.03	0.02	0.03	0.03
	(0.025)	(0.025)	(0.026)	(0.025)	(0.027)	(0.029)	(0.043)	(0.025)	(0.025)	(0.025)	(0.025)	(0.054)
*Eff × Im*		-1.822***										
		(0.305)										
*Eff × FDI*			-3.332***									
			(0.391)									
*Eff × Loc*				-0.42*								
				(0.257)								
*Eff × Size*					-3.852***							
					(0.216)							
*Eff × LP*						-1.232***						
						(0.081)						
*Eff × HHI*							0.0761					
							(0.072)					
*Im× FDI*								-0.28**				
								(0.111)				
*Im× Loc*									0.412***			
									(0.114)			
*Im× Size*										0.5422***		
										(0.088)		
*Im× LP*											-0.152***	
											(0.031)	
*Im× HHI*												-0.004
												(0.061)
*Constant*	-1.202***	-1.322***	-1.332***	-1.392***	-2.222***	-9.412***	-1.202***	-1.202***	-1.152***	-1.182***	-1.482***	-1.202***
	(0.144)	(0.146)	(0.145)	(0.19)	(0.157)	(0.562)	(0.144)	(0.144)	(0.145)	(0.145)	(0.156)	(0.144)
Observations	35,316	35,316	35,316	35,316	35,316	35,316	35,316	35,316	35,316	35,316	35,316	35,316

Source: Authors’ computation

Note: Standard errors in parentheses

* p < 0.1

** p < 0.05

*** p < 0.01

**Table 4:** Export performance model for the medium technology manufacturing industry.

Variable	Model 1	Model 2	Model 3	Model 4	Model 5	Model 6	Model 7	Model 8	Model 9	Model 10	Model 11	Model 12
*Eff*	2.28***	2.99***	2.39***	1.45***	3.93***	11.32***	2.27***	2.28***	2.16***	2.27***	2.13***	2.26***
	(0.245)	(0.295)	(0.267)	(0.42)	(90.347)	(1.78)	(0.246)	(0.245)	(0.246)	(0.245)	(0.248)	(0.245)
*Im*	1.37***	2.62***	1.36***	1.35***	1.36***	1.32***	1.37***	1.35***	0.27*	1.17***	6.40***	1.39***
	(0.080)	(0.301)	(0.080)	(0.080)	(0.079)	(0.080)	(0.080)	(0.087)	(0.154)	(0.131)	(0.649)	(0.080)
*FDI*	0.90***	0.92***	1.32***	0.89***	0.94***	0.95***	0. 90***	0.83***	0.92***	0.91***	0.89***	0.89***
	(0.103)	(0.103)	(0.403)	(0.103)	(0.102)	(0.103)	(0.103)	(0.162)	(0.103)	(0.104)	(0.104)	(0.103)
*Loc*	-1.60***	-1.58***	-1.60***	-2.32***	-1.59***	-1.59***	-1.61***	-1.60***	-1.94***	-1.6***	-1.52***	-1.60***
	(0.08)	(0.080)	(0.080)	(0.306)	(0.080)	(0.080)	(0.08)	(0.08)	(0.081)	(0.08)	(0.081)	(0.08)
*Size*	1.57***	1.57***	1.57***	1.57***	3.36***	1.56***	1.57***	1.58***	1.52***	1.48***	1.48***	1.58***
	(0.075)	(0.075)	(0.075)	(0.075)	(0.278)	(0.075)	(0.075)	(0.076)	(0.076)	(0.090)	(0.076)	(0.075)
*LP*	0.004	-0.005	0.005	0.005	0.0002	0.45***	0.005	0.005	-0.008	0.01	0.13***	0.01
	(0.025)	(0.026)	(0.025)	(0.025)	(0.025)	(0.090)	(0.026)	(0.026)	(0.025)	(0.026)	(0.031)	(0.026)
*HHI*	0.05	0.05	0.06	0.058	0.077	0.12	-0.24	0.05	0.057	0.05	0.044	0.09
	(0.071)	(0.075)	(0.080)	(0.074)	(0.086)	(0.107)	(0.589)	(0.076)	(0.083)	(0.074)	(0.079)	(0.118)
*Eff × Im*		-2.15***										
		(0.498)										
*Eff × FDI*			-0.68									
			(0.631)									
*Eff × Loc*				1.21**								
				(0.505)								
*Eff × Size*					-3.09***							
					(0.46)							
*Eff × LP*						-0.75***						
						(0.15)						
*Eff × HHI*							0.34					
							(0.682)					
*Im× FDI*								0.13				
								(0.209)				
*Im× Loc*									1.44***			
									(0.175)			
*Im× Size*										0.30*		
										(0.161)		
*Im× LP*											-0.41***	
											(0.053)	
*Im× HHI*												-1.53**
												(0.701)
*Constant*	-2.72***	-3.03***	-2.78***	-2.22***	-3.65***	-7.98***	-2.72***	-2.73***	-2.29***	-2.75***	-4.15***	-2.82***
	(0.312)	(0.323)	(0.32)	(0.373)			(0.313)	(0.313)	(0.315)	(0.313)	(0.369)	
Observations	8,244	8,244	8,244	8,244	8,244	8,244	8,244	8,244	8,244	8,244	8,244	8,244

Source: Authors’ computation

Note: Standard errors in parentheses

* p < 0.1

** p < 0.05

*** p < 0.01

**Table 5:** Export performance model for high technology manufacturing industry.

Variable	Model 1	Model 2	Model 3	Model 4	Model 5	Model 6	Model 7	Model 8	Model 9	Model 10	Model 11	Model 12
*Eff*	-1.29***	-1.32***	-1.39***	-1.59**	-1.42***	-1.42	-1.41***	-1.30***	-1.26***	-1.33***	-1.29***	-1.29***
	(0.265)	(0.403)	(0.411)	(0.706)	(0.422)	(2.23)	(0.269)	(0.265)	(0.265)	(0.265)	(0.265)	(0.265)
*Im*	0.72***	0.70***	0.72***	0.72***	0.723***	0.72***	0.73***	0.82***	0.27	1.01***	1.10	0.72***
	(0.082)	(0.235)	(0.082)	(0.082)	(0.083)	(0.082)	(0.082)	(0.115)	(0.234)	(0.124)	(0.765)	(0.082)
*FDI*	1.34***	1.34***	1.27***	1.39***	1.34***	1.34***	1.34***	1.45***	1.35***	1.33***	1.34***	1.34***
	(0.081)	(0.081)	(0.236)	(0.081)	(0.081)	(0.082)	(0.082)	(0.123)	(0.082)	(0.081)	(0.081)	(0.081)
*Loc*	-0.37***	-0.37***	-0.36***	-0.52	-0.37***	-0.37***	-0.37***	-0.38***	-0.66***	-0.35***	-0.36***	-0.36***
	(0.125)	(0.125)	(0.125)	(0.365)	(0.125)	(0.125)	(0.125)	(0.125)	(0.184)	(0.125)	(0.125)	(0.125)
*Size*	0.59***	0.59***	0.59***	0.59***	0.50**	0.59***	0.59***	0.59***	0.59***	0.87***	0.59***	0.59***
	(0.080)	(0.081)	(0.080)	(0.081)	(0.24)	(0.081)	(0.081)	(0.081)	(0.081)	(0.121)	(0.081)	(0.081)
*LP*	0.27***	0.27***	0.27***	0.27******	0.270***	0.264***	0.274***	0.271***	0.266***	0.258***	0.284***	0.269***
	(0.032)	(0.032)	(0.032)	(0.032)	(0.0321)	(0.08)	(0.032)	(0.032)	(0.032)	(0.032)	(0.044)	(0.032)
*HHI*	-0.007	-0.007	-0.0072	-0.007	-0.007	-0.007	-0.17**	-0.007	-0.008	-0.006	-0.007	-0.0003
	(0.011)	(0.011)	(0.011)	(0.011)	(0.011)	(0.012)	(0.068)	(0.011)	(0.011)	(0.011)	(0.011)	(0.013)
*Eff × Im*		0.048										
		(0.487)										
*Eff × FDI*			0.16									
			(0.506)									
*Eff × Loc*				0.33								
				(0.736)								
*Eff × Size*					0.20							
					(0.496)							
*Eff × LP*						0.01						
						(0.166)						
*Eff × HHI*							0.24**					
							(0.096)					
*Im× FDI*								-0.19				
								(0.158)				
*Im× Loc*									0.51**			
									(0.247)			
*Im× Size*										-0.50***		
										(0.162)		
*Im× LP*											-0.03	
											(0.059)	
*Im× HHI*												-0.02
												(0.022)
*Constant*	-4.76***	-4.75***	-4.73***	-4.62***	-4.72***	-4.71***	-4.78***	-4.82***	-4.49***	-4.75***	-4.95***	-4.78***
	(0.394)	(0.405)	(0.404)	(0.498)	(0.41)	(1.038)	(0.395)	(0.397)	(0.414)	(0.396)	(0.552)	(0.395)
Observations	4,104	4,104	4,104	4,104	4,104	4,104	4,104	4,104	4,104	4,104	4,104	4,104

Source: Authors’ computation

Note: Standard errors in parentheses

* p < 0.1

** p < 0.05

*** p < 0.01

In Table 3, our findings suggest that export performance for low-technology manufacturing is positively and significantly affected by technical efficiency, raw material imports, FDI, and firm size. The effect of these variables on export performance is robust to different model estimates and at a high level of significance of less than 1%. There is less strong evidence of the positive effect of industrial concentration on the export performance of low-technology manufacturing. This is because our finding only revealed a positive and significant effect of concentration in two estimates. This finding is supported by the fact that in Indonesia, the concentration of industries is less likely to promote the export of low-technology manufacturing. The observed positive effect of technical efficiency, imports, FDI, size and concentration on export performance is consistent with [48] in the case of the Portuguese wine manufacturing industry [66], in the case of Taiwan [32], in the case of Indonesia, and [67] in the case of German manufacturing firms. Our findings further indicate that low-technology manufacturing industries located in Java Island have lower export performance compared with other firms located in other regions. Productivity of labour asserts a negative and significant effect on export performance. This effect is robust to different model estimates except in Model 5, which shows a positive and significant effect of labour productivity on export performance. The negative effect of labour productivity could be attributed to low technology adoption, which further makes firms labour-intensive with low exportable commodities. Empirical studies have revealed divergent results, which could be a result of reverse causality between export performance and labour productivity [32]. The mixed findings could also result from the use of different proxies of labour productivity [33, 34]. In line with [18]finding firm size has also been found to positively affect export performance in the low-technology industry. This finding corroborates with the fact that the use of a large number of employees as a measure of firm size in the low-technology industry increases Indonesia’s export performance over the study period. The influence of FDI is supported by the fact in Indonesia foreign firms bring new and up-to-date technology for use in manufacturing which further enhances the level of firms’ technology adoption. This also allows for a systematic move from low-technology to medium-technology manufacturing and from medium-technology to high-technology manufacturing.

For the mediation effect on export performance, the findings suggest that in the low-technology manufacturing industry, export performance declines for firms with high technical efficiency and imported raw materials. That is, an increase in technical efficiency while at the same time, importing raw materials will shrink the firm’s export performance in low-technology industries. A negative and statistically significant impact of combined technical efficiency and FDI has been observed in this study. This finding implies that foreign ownership in a highly technically efficient firm using low-technology does not favour export performance. However, high technical efficiency and location, high technical efficiency and firm size, high technical efficiency and labour productivity, high import and FDI, and high import and labour productivity all reduce the export performance of low-technology manufacturing. Our finding indicates that there exists evidence of increased export performance for large firms located in East Java that import high amounts of raw materials. This is because the coefficients of the interacted variables (*Im× Loc* and *Im× Size)* are positive and statistically significant. There exists no significant evidence for increased export performance for technically efficient firms, that import high amounts of raw materials and are highly concentrated within the industrial location.

However, in the case of the medium technology manufacturing industry, in all the estimated models, our findings suggest that there exists robust evidence of the positive and significant effects of technical efficiency, raw material imports, FDI, and firm size on export performance. This is consistent with [18, 40, 47], among others, whereas the positive effect of labour productivity has only been found in two estimates of Models 6 and 11. This result implies that labour productivity can improve capital deepening, which in turn would result in more efficient exports. Additionally, firm location has been found to negatively affect export performance because all the estimated coefficients were negative and statistically significant. This implies that medium-technology manufacturing industries located in Java Island experience low export performance compared to their counterparts in other Islands. This finding contradicts [55].

Our empirical strategy further reports that medium-technology firms that are highly efficient with high raw materials imports, large size, and high labour productivity face declining export performance in Indonesia. Firms with technical efficiency and located on Java Island face an increase in export performance. Our result also indicates that medium-technology firms that import raw materials with larger sizes and are located on Java Island experience an increase in export performance. Firms that import a high amount of raw materials with high labour productivity and industry concentration face a decrease in export performance.

The estimated results show that technical efficiency, location, and concentration of industries reduce export performance for the high-technology manufacturing industry. This finding contradicts [24, 32, 34, 55], among others. The decrease in export performance resulting from industrial location implies that the high-technology manufacturing industry when located on Java Island will experience low export performance compared with similar industries on another island.

Raw material imports, FDI, firm size, and labour productivity promote export performance. This finding is robust to different model estimates. The indirect effect on export performance indicates that firms with high technical efficiency and concentration experience an increase in export performance. Additionally, firms that import a high amount of raw materials and are located within Java Island face high export performance. Export performance decreases for firms with large sizes and imports of high amounts of raw materials. The mediation effect of the analysed variable on export performance is more pronounced in the case of low- and medium-technology manufacturing firms.

From our estimates, we observed that technical efficiency, raw materials imports, FDI, location, firm size, and concentration of industry were significant determinants of export performance in the low-, medium-, and high-technology industries. We observed that technical efficiency promotes exports in the low- and medium-technology manufacturing industries, while in the high-technology industry, it reduces export performance in these manufacturing settings. The decrease in the export of high-technology firms could also be a result of the fact that these firms experience higher local patronage than in foreign markets as they become highly technologically inclined. Additionally, an increase in raw materials imports, FDI, and firm size is associated with increased export performance. This positive impact is a result of the fact that in manufacturing industries, raw imports are mainly used to produce manufactured goods for export. Most FDI is directed toward the export promotion industry, in which case FDI will be associated with increased export and export performance. However, size is an important determining factor of the low-, medium-, and high-technology manufacturing industry in Indonesia. The larger the size of an industry is, the higher the tendency for an expanded industry to produce for export and compete in the international market. Although the number of large firms in both the category of industries is low, but they remained the major firms contributing heavily to Indonesia’s exports. The finding further revealed that location significantly reduces export performance in the low, medium and technology industries. This implies that the geographic nature of Java Island does not seem to favour export performance. We found strong evidence that an increase in labour productivity reduces export performance in the low-technology manufacturing industry. In the case of high-technology manufacturing, an increase in labour productivity promotes export performance. However, in the case of the medium technology industry, there is less strong evidence of the positive effect of labour productivity on export performance. Similarly, the effect of the concentration of industries on exports is less strong in low- and high-technology industries and insignificant in the medium-technology manufacturing industry.

The observed mediation effect of technical efficiency and raw material export has further proven their significant effect on export performance. This is because we found that industries that are technically efficient and import high amounts of raw material experience declining export performance in low- and medium-technology manufacturing. This effect could be because low- and medium-technology firms mostly relied on local raw materials that were less expensive than imported ones. The mediation effect of technical efficiency and raw materials imports affects exports more in the low-technology industry than in medium-technology manufacturing. In the high-technology industry, most of the technical efficiency mediation effects on exports were not statistically significant. These findings imply that technical efficiency and imported raw materials are key determinants of export performance.

## 5. Conclusion

In this study, we scrutinized the issue of export performance in Indonesia’s low-, middle-, and high-technology manufacturing industries over the period 2010–2015. Our empirical strategy revealed a significant result in most of the estimated models. The result indicates that technical efficiency positively affects export performance in the low- and medium-technology industries and reduces export performance in the high-technology manufacturing industry. The reason for the decrease in the export of high-technology firms resulting from increased technical efficiency could be that high-technology manufacturing firms in Indonesia experienced higher patronage in the local market than in the foreign market as they become highly technologically inclined. Furthermore, there exists evidence of the indirect effect of technical efficiency on exports via raw materials imports, FDI, location, size, and labour productivity in low-technology manufacturing. In medium technology manufacturing, the indirect effect of technical efficiency is observed through raw materials imports, location, size, and labour productivity.

High raw materials imports are associated with increased export performance in all categories of manufacturing industries. This is supported by the fact that export and import are closely linked and that most of the materials imported are used to produce for export. The mediation effect of raw materials imports exists through FDI, location, size, and labour productivity in low-technology industries; location, size, and labour productivity; concentration of industries in medium-technology industries; and location and size in high-technology manufacturing industries. The study revealed a significant positive nexus between FDI, size and export performance in all the estimates for the low-, medium-, and high-technology industries. Location asserts a significant negative effect on export performance in all categories of manufacturing industries. This implies that firms located on Java Island are more prone to low export performance. As per the empirical strategy, an increase in labour productivity reduces export performance in the low-technology industry and increases export performance in the medium- and high-technology export industries. However, there is no strong evidence for the effect of industrial concentration on export performance. This is because only in two models we established a positive effect on exports in low-technology industries and a negative effect in one model in the case of the high-technology industry.

Overall, we document that export performance decreases in low- and medium-technology manufacturing with high technical efficiency and imports a significant amount of raw materials. Low-technology industries with high technical efficiency and FDI experience declining exports. Moreover, in low- and medium-technology manufacturing, despite high technical efficiency, larger firms with high labour productivity that are located on Java Island are faced with decreasing exports. Except for firms located on Java Island, low-technology firms that import raw materials and are open for FDI and high labour productivity suffer from declining exports. Export performance increases in medium-technology firms that are large and located on Java Island. Contrary to this finding, in the same medium technology firms, export performance decreases resulting from high labour productivity and the concentration of industries. We found that in high-technology manufacturing, export performance increases in firms that are located on Java Island and import high amounts of raw materials and decreases in large firms that import a large amount of raw materials.

As this study provides insight into the determinants of export performance, the findings obtained from this study have implications for low-, medium-, and high-technology manufacturing industries that are mainly concerned about increasing exports for their manufacturing. Based on the observed positive effect of technical efficiency, low- and medium-technology manufacturing should focus on areas of improving efficiency to promote exports. Since imported raw materials, FDI, and firm size are key to improving exports in low-, medium-, and high-technology manufacturing, a concerted effort is needed by the government to ease any restrictive measure capable of reducing the flow of raw materials imports and FDI. There is also the need for firms to consider the role of size in promoting exports. Location reduces export performance in all categories of manufacturing. There is a need for firms to mitigate the negative effect of location, which can be mitigated by increasing the use of imported raw materials in the case of low- and high-technology manufacturing and efficiency and raw materials imports in the case of medium-technology manufacturing. Additionally, to increase export performance, there is a need to consider simultaneous increases in firm size and import in low- and medium-technology firms, such as import and location in high-technology firms.

### Limitations of the study

The major limitation of this study lies in its focus on Indonesia and its manufacturing industry disaggregated into low-, medium-, and high-technology industries. The empirical findings are therefore manufacturing industry-specific and cannot be generalized to other industries. Future studies should therefore extend this by focusing on agricultural and processing industries while unravelling the low-, medium-, and high-technology agricultural and processing industries. The study also focused on the Indonesian manufacturing industry and there is a need to explore further study of this nature in other countries and regions. Additionally, the study only covered 2010–2015 as there is no available data to go beyond this period. This also served as a major shortcoming of this study. Future work should therefore consider going beyond this period data is made available for researchers by the Central Bureau of Statistics of Indonesia (BPS). Another limitation of this study lies in its focus on the determinants of export performance which cannot all be captured in the formulated model. Future studies should endeavour to provide some insight into other determinants of export performance not considered in this study.

## Supporting information

S1 Appendix (DOCX)

S1 Data (XLSX)

S2 Data (XLSX)

S3 Data (XLSX)
