# Supplementary material for: Determinants of exports performance: Evidence from Indonesian low-, medium-, and high-technology manufacturing industries
Source: PLoS One. 2024 Jan 2;19(1):e0296431. doi: 10.1371/journal.pone.0296431 (PMC10760681; doi:10.1371/journal.pone.0296431)
Supplement: S1 Appendix — (DOCX) [file pone.0296431.s001.docx]

**Appendix A**

**Appendix A1: Efficiency score**

|  | |
| --- | --- |
| **Year** | **Technical efficiency** |
|  |  |
| **Low-Technology** | |
| 2010 | 38 |
| 2011 | 38 |
| 2012 | 38 |
| 2013 | 38 |
| 2014 | 39 |
| 2015 | 39 |
| Average (2010-2015) | 38 |
|  |  |
| **Medium-Technology** | |
| 2010 | 46 |
| 2011 | 45 |
| 2012 | 44 |
| 2013 | 43 |
| 2014 | 43 |
| 2015 | 42 |
| Average (2010-2015) | 44 |
|  |  |
| **High-Technology** | |
| 2010 | 40 |
| 2011 | 36 |
| 2012 | 35 |
| 2013 | 32 |
| 2014 | 29 |
| 2015 | 27 |
| Average (2010-2015) | 33 |
